# Laparoscopic sleeve gastrectomy for a two-and half year old morbidly obese child^☆^

**DOI:** 10.1016/j.ijscr.2013.07.033

**Published:** 2013-09-12

**Authors:** Mohammed Al Mohaidly, Ahmed Suliman, Horia Malawi

**Affiliations:** a Department of Pediatric Surgery, Prince Sultan Military Medical City, Riyadh 11159, Saudi Arabia; b Department of Pediatrics, Prince Sultan Military Medical City, Riyadh 11159, Saudi Arabia

**Keywords:** Laparoscopic sleeve gasttrectomy, Child, Morbid obesity, BMI reduction

## Abstract

**INTRODUCTION:**

Laparoscopic sleeve gastrectomy (LSG) is an accepted technique in bariatric surgery for reducing obesity. Recent reports indicate it to be effective even in children but it has not been tried in very young children.

**PRESENTATION OF CASE:**

We report here a case of a 2 and half years old child subjected to LSG for his morbid obesity and associated obstructive sleep apnea and bowing of legs. LSG was performed after investigations ruled out hereditary or genetic causes of obesity. The procedure was well tolerated without any complications and 2 months post surgery, the obstructive sleep apnea decreased substantially. The child was followed up for two years. At the last follow up BMI was drastically reduced from a pre surgical value of 41.1–24 kg/m^2^ at 24 months post surgery.

**DISCUSSION:**

Prior to our report the youngest child to undergo sleeve gastrectomy was 5 years old from Saudi Arabia. We observed LSG to be safe and effective in reducing obesity and related co morbidities in a two and half year's old child.

**CONCLUSION:**

The results suggest that LSG can be a safe and effective alternative for weight control in morbidly obese children even of less than 3 years of age. However more studies and long term follow up is essential for monitoring the growth and development of children subjected to LSG.

## 1 Introduction

Obesity among children is a worldwide problem.^1^ It is not only associated with preventable long term health conditions such as hypertension, type 2 diabetes mellitus (DM), dyslipidemia, obstructive sleep apnea (OSA), and orthopedic complications but also results in serious psychosocial complications.^2,3^ Recent studies suggest that obesity and overweight even in children less than two years of age is associated with early morbidity.^4^ Overweight and obese toddlers (<2 years of age), as compared to normal weight children were observed to have more admissions as well as a larger number of repeated admissions, development delays and high incidence rates of respiratory morbidity such as snoring, asthma and stridor.^4^ Therefore reducing obesity in young children is essential to reduce morbidity and also since obesity during childhood leads to obesity in adulthood.^5^

Severely obese children with co morbidities and those who do not respond to life style changes and pharmacotherapy get benefit from bariatric surgery.^6^ Laparoscopic Sleeve Gastrectomy (LSG) is gaining acceptance for the treatment of morbid obesity under several indications.^7–10^ As LSG proved to be effective in the short-term in achieving considerable weight loss, it has been proposed by some to be used solely as a bariatric procedure. Recent reports show that morbid obesity and the associated co morbidities were successfully managed by LSG even in children of 5 and 6 years of age.^5,6,11,12^ However to our knowledge LSG has never been tried in very young age children.

We present here probably the first case report of the successful management of a two and a half year old morbidly obese boy with sleep apnea and bowing of legs with laparoscopic sleeve gastrectomy.

## 2 Case report

**Fig. 1:**
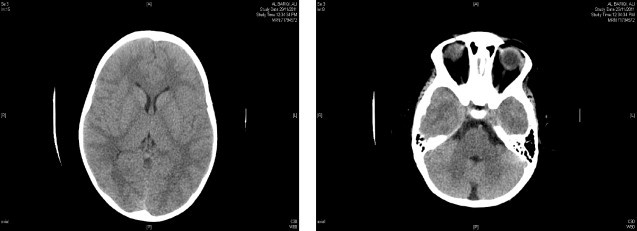
Computed tomography of the brain.

This two-year-and-a-half old boy was referred from the pediatric endocrinologist as a case of morbid obesity for surgical assessment and management. His parents’ main complaint was an abnormal increase in weight, snoring and apneoic attacks during sleep requiring them to closely monitor his sleep. He was a product of an uneventful delivery with a birth weight of 3 kg. He was breast fed for 2 months and continued on formula milk up to one year of age. Supplementary food was introduced at 9 months (as father mentioned). He was well until 6 months of age, when the parents noticed he was looking chubby and overweight, his weight was 9.7 kg; they sought medical advice at 8 months of age. At presentation to the endocrinologist he was 14 months of age with a weight of 21.3 kg (BMI = 29 kg/m^2^). He was started on diet and medical management and all his hormonal and chromosomal work up was normal and there was no change in the milestones. The patient had no family history of morbid obesity or genetic abnormality. CT scan of the brain also showed no other causes of obesity such as pituitary or cerebellar tumor (Fig. 1). All attempts to control weight increase through diet and medical control failed and within 4 months his weight increased by eight kilograms (29.3 kg, BMI = 33 kg/m^2^). Although the parents were informed about the importance of a strict dietary regimen a full compliance cannot be ascertained mainly due to the different socio cultural habits and the absence of the practice of calculating the calorific value of the diet.

**Fig. 2:**
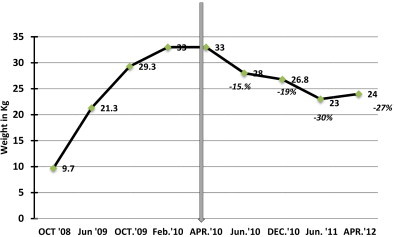
Body weight pre and post LSG.

**Fig. 3:**
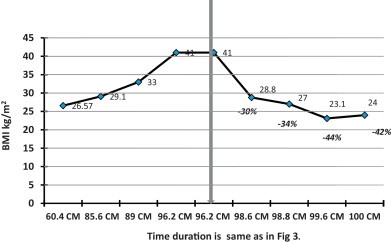
Body mass index pre and post LSG.

The increase in weight was associated with obstructive sleep apnea symptoms and bowing of the legs. When he was presented to the surgical clinic, he was 29.4 kg with a BMI of 36 kg/m^2^ and he was on 12.5 mcg of l-thyroxin. The patient had another trial of medical management and was referred to the obesity clinic for further dietary management. However, his obstructive sleep symptoms instead of improving showed an increase in frequency and after 18 months of therapy (weight = 33 kg, BMI = 41 kg/m^2^) (Figs. 2 and 3), he was admitted for a LSG which was performed in April 2010. Due to an increase in the episodes of apnea and also due to the difficulty in intubation preoperative endoscopy could not be performed.

**Fig. 4:**
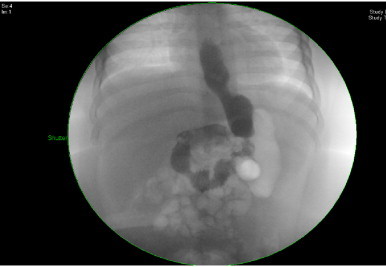
Post-operative day 5 upper GI contrast study.

A 32 F bougie was used and gastrectomy was started 3 cm proximal to pylorus and carried up to 15 cm lateral to angle of his and about 1 cm away from the bougie to avoid tension on the suture line. The gastric sleeve was closed using Eshelon staple (60 mm), and was reinforced with Seam guard. After the occlusion, insufflations of the stomach were performed using methylene blue and no leak was detected. Post operative follow up also showed no leaks or bleeding. Low-molecular-weight heparin (LMWH) was initiated after surgery and discontinued after he started adequate mobilization on day 3 post surgery. Post operative day 5 contrast study of the upper GI tract showed normal opacification of the remaining stomach and no leak from anastomoses site (Fig. 4). The patient was started orally on liquids five days post-operation and progressed to pureed diet. Post surgery, the child was put on anti reflux medication and was evaluated with an upper GI study after three months and the medication was stopped. However no complain of reflux was observed during this period and also during the subsequent follow ups.

**Fig. 5:**
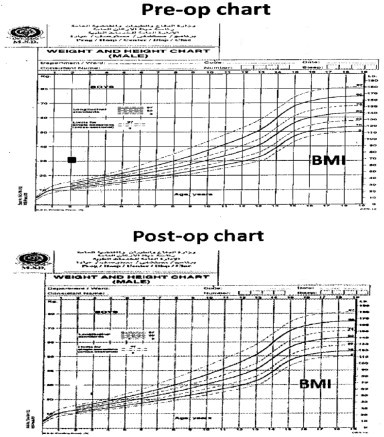
Pre and post operative BMI Charts.

A reduction of 15% in body weight and 30% in BMI was observed at 2 months follow up post LSG and the obstructive sleep symptoms were considerably resolved. At the last follow up, 24 months after LSG, a considerable reduction in the body weight (27%; from 33 kg to 24 kg) and BMI (41.46%; from 41 kg/m^2^ to 24 kg/m^2^), was recorded (Figs. 2 and 3). One of the limitations of this report is that the parents of the child did not comply with the provided instruction/s and more often showed a tendency to miss appointments and hence a regular time bound follow up was not possible (Fig. 5). Normalization in the pre surgical high levels of TSH and triglycerides was observed at six months post LSG. Full blood count, thyroid function test, liver function test, kidney function test, blood glucose, cortisol, growth hormone and blood electrolytes were all within normal limits at the last follow up at 24 months post LSG (Tables 1 and 2).

## 3 Discussion

In recent years there has been renewed interest in the surgical treatment of morbid obesity in concomitance with the epidemic of obesity and application of the laparoscopic techniques to the field of bariatric surgery as well.^14^ Among the different approaches of bariatric surgery, LSG is being used both as a staged and stand alone procedure in adults.^13^ Bariatric surgery has also been suggested to result in sustained and clinically significant weight loss in pediatric patients with morbid obesity but with a potential for serious complications.^14^ However the importance of bariatric surgery for reducing obesity in children is being increasingly realized.^6,11,15,16^

We observed LSG to be safe and effective in reducing obesity and related co morbidities in the two years and 6 months old child. The procedure was well tolerated and the child was followed up for 24 months without any complications and normal milestones. Prior to our report the youngest child to undergo sleeve gastrectomy was a 5 years old from Saudi Arabia.^6^

Data from other recent studies also show a positive outcome in children undergoing LSG surgery for morbid obesity and the associated co morbidities. Till et al.^12^ performed LSG in a pediatric series of 4 patients with a mean age of 14.5 years and at 12 months follow up no complications including malnutrition or vitamin deficiency were observed. Furthermore a significant improvement in the associated co morbidities such as Type 2 diabetes, hypertension etc. was observed. An earlier study has also shown a reduction in body weight and related co morbidities in a 6 years old child following LSG.^17^ A very recent review of LSG performed on 108 obese children and young adults by a single surgeon in Saudi Arabia also showed LSG to be fairly effective in short term weight loss in more than 90% of pediatric patients and resolution of 70% of co-morbid problems associated with obesity.^6^ Beneficial effects of LSG in the form of reduction of BMI from 42 kg/m^2^ to 28 kg/m^2^ and resolving of knee pain were also observed in a 10 years old child suffering with morbid obesity and Blount's disease (tibia vara).^10^ A more recent study also showed the successful reduction of morbid obesity and associated obstructive sleep apnea, acanthosis nigricans and hypertension by LSG in a young patient.^8^

Besides weight loss and metabolic benefits, bariatric procedures including LSG has wider implications and are associated with improving the quality of life by reversing/reducing several obesity related co morbidities such as obstructive sleep apnea.^18^ We observed that the problem of obstructive sleep apnea was considerably reduced within 2 months of the procedure. Earlier studies both in adults and children have also shown a reduction in the prevalence of obstructive sleep apnea in obese patients following bariatric surgeries.^6–8,19,20^

Serum proteomic and metobonomic profiling after LSG in children and adolescents has revealed that it leads to changes in amino acids and in lipid metabolism and decrease in obesity related biomarkers.^21^ These biomarkers will help in providing measures for monitoring therapy and the induced physiological changes following LSG.^21^

The reduction in the BMI and the associated co morbidities in the child post gastrectomy, lack of complications at two years follow up and normal milestones suggest that gastrectomy is safe and can be performed in young children to reduce BMI and the associated co morbidities. However a potential disadvantage of LSG is its irreversibility and the lack of proper understanding of the consequences by the young children. Therefore a very honest and open discussion with the parents regarding the permanence and irreversible nature of the procedure is essential during the decision making process.

## 4 Conclusion

LSG may be used in very young children provided they have co-morbidities and no improvement with medical and conservative multidisciplinary management. In our patient, the weight reduction was significant and his associated symptoms resolved with time indicating its safety and efficacy.

## Conflict of interest

The authors declare that there is no conflict of interest in undertaking this study.

## Funding

None.

## Ethical approval

Written consent was obtained from the patient for publication of this case report and accompanying images. A copy of the written consent is available for review by the Editor-in Chief of International Journal of Surgery Case Reports.

## Author contributions

All the authors have contributed in this study. Dr. Al Mohaidly and Dr. Suliman were involved in carrying out the surgery and follow up. Dr. Mawlawi was involved in the medical management of the patient before and after surgery and the follow up. All the authors were involved in data collection and analysis.

The authors acknowledge Dr. Abdulrahman Al Asmari for his support and review of the manuscript.

## Figures and Tables

**Table 1:** Preoperative investigations.

Parameters	Level/s
FBS	5.8 mmol/L
HBA1C	5.6 mmol/L
Cholesterol	4.01
Triglyceride	2.40 high
FT4	16.4 (12–22) pmol/L
TSH	7.080 H (0.27–4.2)
25 Hydroxy Vitamin D	33.3 L
Ferritin	15 L
Iron	5 L
T.I.B.C	59 N
Corrected calcium	Normal
Inorganic phosphate	Normal
Alkaline phosphatase	Normal
Prolactin	Normal
Cortisone	Normal
GH	Normal
Serum insulin	Normal
C-peptide	Normal
Calcium	Normal
CBC	Normal
U&E	Normal
LFTs	Normal

**Table 2:** Post operative investigations.

	2010 October	2011 March	2011 October	2012 March
FBS	Normal			
Ferritin	20 L	Normal		
U&E	Normal			
LFTs				
Cholesterol				
Triglyceride				
FT4				
TSH				
Prolactin				
Cortisone				
GH				
Iron		Normal		
25 Hydroxy Vitamin D				
Calcium				
T.I.B.C				
	
Corrected calcium				
Inorganic phosphate				
Alkaline phosphatase				
